# The combined effects of anthropogenic and climate change on river flow alterations in the Southern Caspian Sea Iran

**DOI:** 10.1016/j.heliyon.2024.e31960

**Published:** 2024-05-29

**Authors:** Alireza Sharifi, Aziza Baubekova, Epari Ritesh Patro, Björn Klöve, Ali Torabi Haghighi

**Affiliations:** Water, Energy, and Environmental Engineering Research Unit, University of Oulu, P.O. Box 8000, Oulu, Finland

**Keywords:** Anthropogenic activities, Climate change, Budyko framework, Socio-Hydrology, River flow alteration, Caspian sea

## Abstract

In recent years, the effects of human activities and climate change on river flow patterns have become a major concern worldwide. This is particularly true in the southern Caspian Sea (SCS) region of Iran, where increasing water-intensive socio-economic development and climate change have significantly altered river flow regimes. To better understand these changes, this study employs two nonparametric methods, the modified Mann-Kendall method (MK3) and Innovative Trend Analysis (ITA), to examine spatial and temporal changes in hydrometeorological variables in the SCS. The study also evaluates the impact of human activities and climate change on river flow alteration using elasticity-based methods and the Budyko hypothesis in 40 rivers on the closest gauges to the Caspian Sea. The results indicate an alarming trend of increasing temperature, potential evapotranspiration, and decreasing river flows in the SCS region. In particular, human activities were found to be responsible for around 91.7 % of the change on average, resulting in a significant decline in inflow to the Caspian Sea by about 3216 MCM annually. This declining trend in inflow could potentially exacerbate the eutrophication conditions in the Sea and negatively impact its ecosystem and economics. Therefore, appropriate measures need to be taken to address these environmental and socio-economic issues in the southern Caspian Sea region.

## 1 Introduction

Rivers are one of two primary sources for supplying water to meet agricultural, domestic, industrial, and environmental demands [1]. However, the combined impacts of anthropogenic activities and climate change are threatening the river-connected ecosystems and societies in various regions and climate systems [[2], [3], [4]]. Anthropogenic activities such as land use change, urbanization, deforestation, desertification, and reservoir operation have altered rivers' flow regimes and flow characteristics. For example, hydropower activities shift peak flow timing and magnitude [5,6], and overusing surface water and unsustainable development decreases the discharge [[2], [7], [8]]. Moreover, these activities can influence basin characteristics, including the rate of infiltration, the balance of evapotranspiration, and soil moisture. This, in turn, increases the risk of erosion and sedimentation [9], negatively impacting the ecosystem [10]. On the other hand, climate change, climate variability, and global warming can significantly contribute to alterations in river flow regimes by elevating temperatures and altering spatiotemporal precipitation patterns [11]. The distinction between climate variability and climate change lies in their temporal scales, with climate variability encompassing changes occurring within shorter timeframes. Therefore, an essential step toward enhancing, developing, and modifying sustainable watershed management, preserving the ecosystem functioning of streams, and formulating effective climate change adaptation policies is the assessment and quantification of the impact of anthropogenic activities and climate change on alterations in river flow regimes [12].

In recent years, a variety of approaches have been applied to assess and quantify the impact of anthropogenic activities and climate change on river flow alteration, including empirical methods [13,14], elasticity-based methods [15,16], and hydrological modeling [17,18]. Empirical methods, which are generally the most straightforward approach, have three limitations specifically: (1) the necessity of long-term hydrological and precipitation data; (2) ignoring the role of other variables in flow regime alteration such as temperature and evapotranspiration; and (3) ignoring the physical mechanism of runoff generation. Alternatively, elasticity-based methods and hydrological models have a more physically realistic basis and estimate river flow using more variables in the runoff generation process [19]. Physically-based hydrological models usually follow a time-consuming, costly, and complex approach and require a great deal of data, some of which may be difficult to obtain. Hence, elasticity-based methods are the fastest and most intuitive methods to assess the impacts of anthropogenic activities and climate changes on river flow alteration [16].

The Caspian Sea is the largest continental water body on the earth (almost 40 % of the world's continental surface water) [20] and plays an essential role in meeting environmental demands and providing ecosystem services. This water body, with a water volume of around 78,000 km^3^, covers an area of about 390,000 km^2^ in five countries Iran, Russia, Azerbaijan, Kazakhstan, and Turkmenistan. Nine sub-basins around the sea provide about 300 km^3^ of fresh water to the Sea every year [21]. With about 6380 km of coastline, this Sea is receptive to about 11 million people on its coast, most of whom live in Azerbaijan and Iran [[22], [23], [24]]. Over the last decades, anthropogenic activities such as agriculture, industry, urbanization, transportation, and fishing have expanded throughout the Caspian Sea and around its coast, which has substantially affected water quality and increased environmental degradation of the Sea [23]. On the other hand, the Caspian Sea level has undergone dramatic fluctuations over the last decades and decreased substantially by about 6.8 cm/yr from 1996 to 2015 due to declining inflow to the Sea and increasing evaporation rate [25]. By 2100, the sea level is projected to decrease by 8 and 10 m under the RCP4.5 and RCP8.5 climate change emission scenarios, respectively [26]. Consequently, reducing the Caspian Sea level can boost eutrophication conditions resulting in socioeconomic and ecological problems.

The southern Caspian Sea (SCS) in the north of Iran with relatively compact vegetation and mild weather is an attractive and beautiful region for farming, settlement, tourism, and socio-economic activities [27]. This region had the most rain-fed agricultural production in Iran between 2004 and 2013 [28] and the mean annual harvest of bony fish was about 17,700 tons from 1997 to 2008 [29]. Due to the distinct climate, this region has always been exciting for investors and tourists, it attracted over 33 million tourists in 2015 [30]. In recent years, there has been an increasing rate of migration from other parts of Iran, especially Tehran (the capital of Iran) to this region, because of increasing environmental problems such as air pollution, land subsidence [31], and dust storms [32]. Growing demand for housing and rapid urbanization in this region have led to farmlands transforming into urban and industrial lands, while the forests located in the middle of the region are being replaced with agricultural lands [23]. The SCS also is a trade window for Iran to northern neighbor countries such as Russia and Kazakhstan. Therefore, socioeconomic development in this region has been incrementally followed, especially in the last two decades. It can result in increased water withdrawal and severely affect the water supply needs of various sectors.

This paper makes a significant contribution to quantifying river flow alteration by modifying the elasticity-based methods in an extended region. For this purpose, the trend of hydro-meteorological variables in the study area, including temperature, potential evapotranspiration (PET), precipitation, and river flow, is being investigated. Additionally, due to variations in the length of river flow data, we examine different periods to identify significant change points in the time series of river flow. This approach allows for a more comprehensive comparison of human activities and the impacts of climate change across various periods. Then, the impact of anthropogenic and climate change on rivers' flow alteration is assessed using the Budyko hypotheses, and the volumetric change in the rivers is characterized. Finally, the consequences of rivers' flow alteration in the SCS and its effects on the socioeconomic development path in this region are discussed. While this study provides valuable insights into the impact of human activities and climate change on river flow alteration, it is important to acknowledge its limitations, including the type and length of data.

### 1.1 Study area and data set

**Fig. 1:**
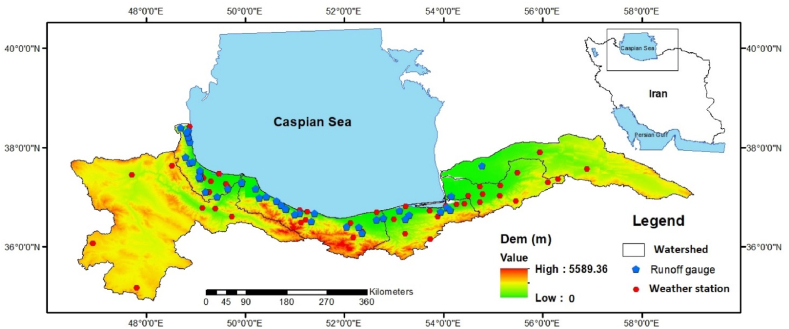
South part of the Caspian Sea in the north of Iran. The spatial distribution of the weather stations (red circle) and the runoff gauges (blue pentagon) are shown in this figure.

The SCS in Iran covers an area of about 159,000 km^2^, in six sub-basins, including Atrak, Gorgan-Rud, Haraz-Neka, Lahijan-Noor, Sefid-Rud, and Talesh. These sub-basins supply about 15 km^3^ of fresh water to the Caspian Sea annually (Fig. 1). The annual precipitation varies from about 250 to 1700 mm from the east to west of the region. Based on the Köppen-Geiger climate classification [33], this region is influenced by eight climate types, most of which are BSk (arid, steppe, cold), followed by BWk (arid, desert, cold) and Csa (temperature, dry summer, hot summer). About 117 dams have been constructed on the rivers across the SCS sub-basins, which regulate more than 5 BCM of water annually. Sefid-Rud Dam, with a capacity of 1800 million cubic meters (MCM), is the biggest dam in this region that went into operation in 1962.

In this study, monthly flow data from 40 gauges (the closest gauges to the Caspian Sea) in 40 rivers and monthly precipitation data from 36 weather stations were used (Fig. 1). The flow data were obtained from the Iran Water Resources Management Company (IWRMC), while the precipitation data were collected from IWRMC and the Iran Meteorological Organization (IRIMO). The oldest flow data in the study region has been recorded since 1950 in the Babol-Rud, Haraz, Chaloos, and Cheshmeh Kileh rivers at Babol, Karehsang, Pole Zoghal, and Haratbar gauges, respectively. Therefore, the length of flow data in selected gauges differed from 22 to 67 years because the monthly flow data were investigated from its establishment to 2017.

In addition to considering flow and precipitation, the assessment of anthropogenic activities and the contribution of climate change to river flow alteration requires the use of PET, evaluated through elasticity-based methods. Hence, the maximum and minimum temperature data from 35 stations were employed to calculate PET using the modified Hargreaves model [34]. Monthly temperature data from these 35 stations were sourced from the IWRMC and IRIMO. Due to the limited data length in most synoptic stations within the study region and the absence of sunshine hours data in some locations, only temperature data from select synoptic stations were utilized.

## 2 Methods

**Fig. 2:**
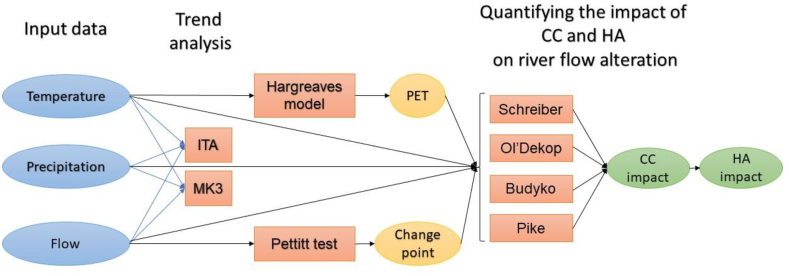
Path of quantifying the impact of human activities (HA) and climate change (CC) on river flow alteration.

In this study, the elasticity-based methods and Budyko hypotheses were applied to assess anthropogenic activities and climate change's contribution to river flow alteration. These methods were set up for rivers' flow time series in which the Pettitt test [35] reveals a significant change point. One of the requirements for elasticity-based analysis is PET. The modified Hargreaves model [34] was used to estimate PET. In addition, the overall spatial/temporal change in temperature, precipitation, PET, and flow across the region is evaluated. To assess spatial/temporal change, two non-parametric methods, including the modified Mann-Kendall method (MK3) [36] and Innovative Trend Analysis (ITA) [37] were applied to analyze the trend of different parameters. Hamed and Rao [36] made modifications to the widely employed nonparametric Mann-Kendall (MK) method [38,39]. These adjustments were designed to eliminate any significant autocorrelations present in the time series data, which typically exhibit autocorrelations at different time lags. The presence of autocorrelation in hydro-meteorological data can significantly influence the results of trend analysis, necessitating its consideration for the accurate identification of a monotonic trend and its significance [40]. Furthermore, ITA [37], a straightforward graphical approach for examining time series trends, was integrated. ITA is particularly suitable for shorter data sets and does not share the limitations of the MK method, such as assumptions about the independence of time series structure and the normality of the distribution. The different stages of the current study are shown in Fig. 2.

### 2.1 Hargreaves model for PET analysis

The modified Hargreaves model [34] is developed based on [41] and is widely used in various climate regions [42]. This model used minimum and maximum temperatures to estimate PET. The proposed form of this model is as follows [34]:

(1) $${ET}_{p}=0.0023R_{a}\left(T_{ave}+17.8\right){\left(T_{\max}-T_{\min}\right)}^{0.5}$$

where ${ET}_{p}$ is PET (mm day^−1^), $R_{a}$ is the water equivalent of extraterrestrial radiation (mm day^−1^), $T_{ave}$, $T_{\max}$, and $T_{\min}$ are the mean, maximum, and minimum air temperature (°C), respectively.

### 2.2 Modified Mann-Kendall

This approach is based on the nonparametric Mann-Kendall (MK) method [38,43], which is widely applied for the identification of trends in the hydrologic and climatologic time series [44]. Hamed and Rao [36] developed this approach to remove all significant autocorrelations in the time series data.

Assuming that the data are independent and identically distributed, the *S* statistic of the MK test is computed using eq. (1) [38,39]:

(2) $$S=\sum_{i=1}^{n-1}\sum_{j=i+1}^{n}\text{sgn}\left(x_{j}-x_{i}\right)$$

where x_*i*_*,* and x_*j*_ denote sequential *i*th and *j*th data points, respectively; *n* denotes the sample size; and sgn (.) is the sign function which can be computed as:

(3) $$\begin{array}{cc} \text{sgn}\left(\theta \right)=\left\{ \begin{array}{ccc} 1 & if & \theta >\circ \\ \circ & if & \theta =\circ \\ -1 & if & \theta <\circ \end{array}\right.\text{,} & \theta =\left(x_{j}-x_{i}\right) \end{array}$$

The mean (E(S)) and variance (Var(S)) of the *S* statistic in Eq. (2) are as follows [39]:

(4) E(S)=∘

(5) $$V\left(S\right)=\frac{n\left(n-1\right)\left(2n+5\right)-\sum_{i=1}^{n}t_{i}\left(t_{i}-1\right)\left(2t_{i}+5\right)}{18}$$

where *t*_*i*_ denotes the number of data points in the *t*th group.

In this new approach, a modified variance of S (V(S)*) computes as follows [36]:

(6) $${V\left(S\right)}^{*}=V\left(S\right)\frac{n}{n^{*}}$$

where n* is the effective sample size. The $\frac{n}{n^{*}}$ ratio can be calculated as follows [36]:

(7) $$\frac{n}{n^{*}}=1+\frac{2}{n\left(n-1\right)\left(n-2\right)}\sum_{i=1}^{n-1}\left(n-i\right)\left(n-i-1\right)\left(n-i-2\right)r_{i}$$

where *r*_*i*_ denotes the lag-*i* significant autocorrelation coefficient of rank *i* of time series.

Then the standardized statistic of the *S* statistic i.e. *Z* can be computed as:

(8) $$Z=\left\{ \begin{array}{cc} \frac{S-1}{\sqrt{{V\left(S\right)}^{*}}} & S>\circ \\ \circ & S=\circ \\ \frac{S+1}{\sqrt{{V\left(S\right)}^{*}}} & S<\circ \end{array}\right.$$

If the computed *Z* is greater than ${-Z}_{1-\alpha /2}$ or less than $Z_{1-\alpha /2}$ , there is no trend in the time series at the significance level of α. On the other hand, if *Z* value is positive and greater than $Z_{1-\alpha /2}$ , the trend is upward whereas if the *Z* value is negative and less than ${-Z}_{1-\alpha /2}$ , the trend is downward. The MK Z is tested for the significance of trend based on different threshold levels, i.e., 1.645 for 10 %; 1.96 for 5 %; and 2.33 for 1 % confidence level.

### 2.3 Innovative trend analysis

The second non-parametric test, the ITA, does not have the restrictive assumptions of the MK test, such as the assumption of the independent structure of the time series and normality of the distribution [37]. In this method, the data set splits into two equal parts to plot the first half of the time series against the second half [37]. If all the points fall on the 1:1 line, there is no trend in the time series. Conversely, there is an increasing (decreasing) monotonic trend in the time series if all the points are located above (below) the 1:1 line. A monotonic trend means that the variable changes consistently through time (i.e., increases or decreases), but the trend may or may not be linear. The time series has nonmonotonic trends when the points do not entirely fall above (below) the line and instead intersect the 1:1 line [45].

### 2.4 Pettitt test

The Pettitt test [35], as a non-parametric test, is based on the Mann-Whitney two-sample test (rank-based) that has been used to detect abrupt changes in the mean of the distribution of the variable of interest [46]. The null hypothesis H_0_ is no change in the distribution of a sequence of random variables when arbitrarily splitting the sample in two [47]. The Pettitt test produces a ranked-based comparison between two observations before and after a date t through the so-called Pettitt statistic ($U_{t,T}$), which can be calculated as:

(9) $$U_{t,n}=\sum_{i=1}^{t}\sum_{j=t+1}^{n}sgn\left(x_{j}-x_{i}\right)$$

where n is the size of the variable. The Pettitt statistic is assessed for all random variables from 1 to n. Then, the most significant abrupt change is selected where the statistic has the greatest absolute value:

(10) $$K_{n}=\max_{1\leq t<n}\left|U_{t,n}\right|$$

A change point occurs at time t when the statistic $K_{n}$ is significantly different from zero at a given level. The significance probability associated with the rejection of H_0_ is approximated by:

(11) $$p=2.\exp\left(\frac{-6K_{n}^{2}}{n^{2}+n^{3}}\right)$$

Once the p-value is less than the significance level of 0.05, we can reject the null hypothesis and divide the data into two sub-series (before and after the change point time).

### 2.5 Quantifying the impact of anthropogenic activities and climate change on river flow alteration

It is widely accepted that river flow alteration results from anthropogenic activities and climate change. Hence, the total change in annual mean flow (AMF) ($\Delta \bar{Q}$) can be defined as [48]:

(12) $$\Delta \bar{Q}=\Delta {\bar{Q}}_{ha}+\Delta {\bar{Q}}_{cc}$$

where, $\Delta {\bar{Q}}_{ha}$ and $\Delta {\bar{Q}}_{cc}$ are the changes in AMF due to anthropogenic activities and climate change, respectively.

The change in AMF is calculated by comparing the AMF in two specific periods, before and after the time of abrupt change. These two periods were introduced as baseline and variation periods based on detected abrupt change through the Pettit test. Accordingly, the AMF change (ΔQ) is the difference between AMF in baseline and variation periods (Eq. (13)) which is equal to the sum of the contribution of anthropogenic activities and climate change (Eq. (12)).

(13) $$\Delta \bar{Q}={\bar{Q}}_{var}-{\bar{Q}}_{bl}=\Delta {\bar{Q}}_{ha}+\Delta {\bar{Q}}_{cc}$$

where, ${\bar{Q}}_{var}$ and ${\bar{Q}}_{bl}$ are the AMF in the variation and baseline periods, respectively.

### 2.6 Elasticity-based methods and Budyko hypotheses

According to Ref. [16], runoff (Q) in a basin can be expressed as the following function:

(14) $$Q=f\left(P.{ET}_{p}.V\right)$$

where P is precipitation, ${ET}_{p}$ is PET, and V is the characteristics of the basin. Changes in runoff (ΔQ) can be expressed as follows:

(15) $$\Delta Q=\frac{\partial Q}{\partial P}\Delta P+\frac{\partial Q}{\partial {ET}_{p}}\Delta {ET}_{p}+\frac{\partial Q}{\partial V}\Delta V$$

where ΔP and $\Delta {ET}_{p}$, are the change in mean annual precipitation and PET, respectively, and ΔV is the change in basin characteristics. Any change in climate variables is due to the impact of climate change on precipitation and PET. Therefore, the contribution of runoff change due to climate change can be defined as follows:

(16) $${\Delta \bar{Q}}_{cc}=\frac{\partial Q}{\partial P}\Delta P+\frac{\partial Q}{\partial {ET}_{p}}\Delta {ET}_{p}$$

By replacing the climate elasticity of runoff elasticities of runoff to precipitation ($\varepsilon_{p}=\frac{\partial Q/Q}{\partial P/P})$ and PET ($\varepsilon_{{ET}_{p}}=\frac{\partial Q/Q}{\partial ETp/ETp}$) in Eq. (16), The contribution of climate change in runoff (according to Schaake, 1990) can be expressed as:

(17) $${\Delta \bar{Q}}_{cc}=\left(\varepsilon_{P}\Delta P/P+\varepsilon_{{ET}_{p}}\Delta {ET}_{p}/{ET}_{p}\right)Q$$

where $\varepsilon_{P}$ and $\varepsilon_{{ET}_{p}}$ are the climate elasticities of runoff to precipitation and PET, respectively.

According to the long-term water balance, runoff can be expressed as follows:

(18) $$Q={P-ET}_{a}-\Delta S$$

where, ${ET}_{a}$ is the actual evapotranspiration, and ΔS is the change in water storage which can be assumed to be zero on a multi-year scale. Budyko (1974) defined two indexes, including the aridity index (φ), which is the ratio of the PET to the precipitation (Eq. (19)), and the evaporative index (F(φ)), which is the ratio of the actual evapotranspiration and to the precipitation (Eq. (20)). The relationship between these two indexes as known as Budyko curve. For more information about the Budyko curve, please refer to Ref. [49].

(19) $$\varphi ={ET}_{p}/P$$

(20) $${ET}_{a}=PF\left(\varphi \right)$$

A combination of the climate elasticity of runoff with Eq. (18), the precipitation elasticity coefficient, and the PET elasticity coefficient for runoff can be derived as:

(21) $$\varepsilon_{P}=1+\frac{\varphi F՛\left(\varphi \right)}{1-F\left(\varphi \right)},\varepsilon_{P}+\varepsilon_{{ET}_{p}}=1$$

where, F՛(φ) represents the derived function of F(φ). For more details about the derivation of $\varepsilon_{P}$ please refer to Ref. [19]. Different methods were applied to estimate F(φ). In this study, four different equations [[50], [51], [52], [53]] were used to estimate F(φ) (Table 1).

**Table 1:** Different forms of the evaporative index F(φ).

Function	F(φ)
Schreiber [50]	$F\left(\varphi \right)=1-e^{-\varphi }$
Ol’Dekop [49]	F(φ)=φtanh(1/φ)
Budyko [54]	$F\left(\varphi \right)={[\varphi \;\tanh\;\left(1/\varphi \right)(1-e^{-\varphi })]}^{1/2}$
Pike [55]	$F\left(\varphi \right)=1/\sqrt{1+\varphi^{-2}}$

## 3 Results

### 3.1 Hydro-meteorological variables trends

**Fig. 3:**
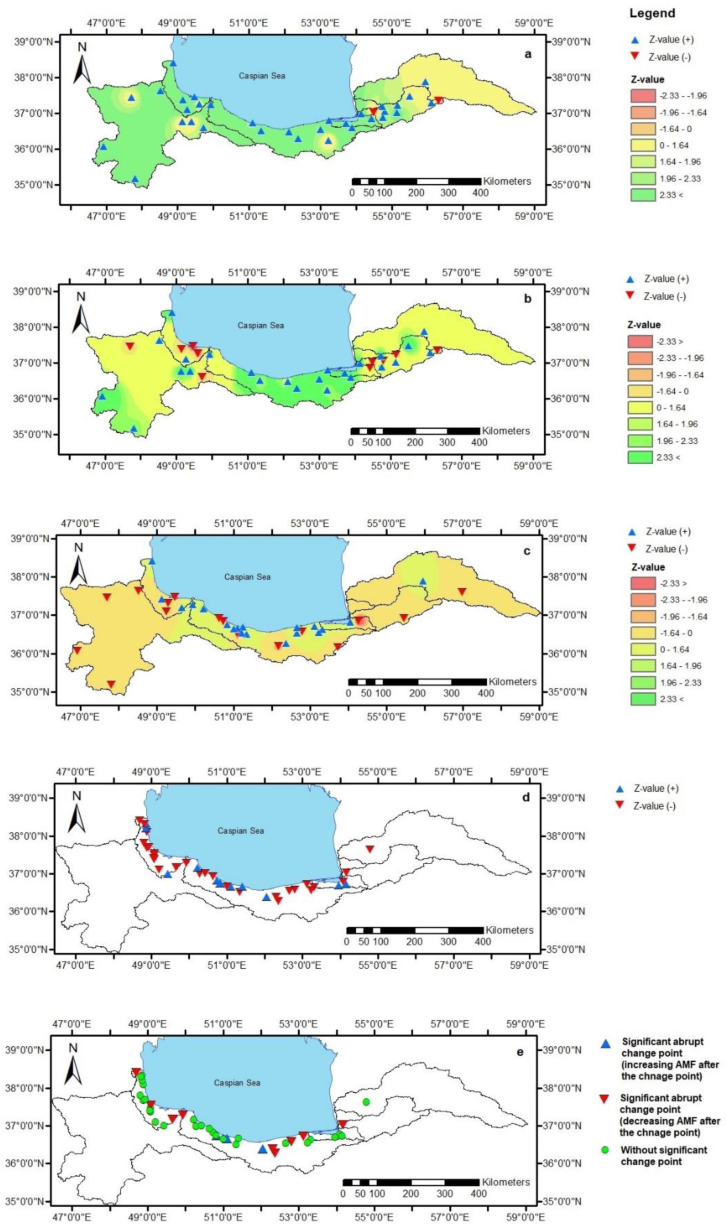
Z-value and its spatial distribution obtained from MK3 for temperature (a), evapotranspiration (b), and precipitation (c). (d) shows the Z-value of the annual mean flow at the gauges. (e) illustrates the gauges in which a significant abrupt change point has been found in the time series using the Pettitt test.

The temperature has increased almost in all the study regions, and this increase has been more significant in the west and central regions of the study area (Fig. 3a). The temperature in 19 and 4 (of 35) stations has increased substantially at 1 % and 5 % confidence levels, respectively, while for one station (Agh Ghala), it declined at a 5 % confidence level (Table 2 and Fig. 3a). Likewise, the PET has increased in all the stations in the center of the study area, and this increase has been significant. However, it displayed a negative trend in several stations in the southwest and southeast of the Caspian Sea (Fig. 3b). The PET has substantially increased in 14, 3, and 1 (of 35) stations at 1 %, 5 %, and 10 % confidence levels, respectively. In addition, it has declined significantly in two stations (Rasht and Bandar Anzali) at a 1 % confidence level (Table 2).

**Table 2:** The trend of temperature and evapotranspiration based on the Mann-Kendall.

Name	Temperature		Evapotranspiration	
	Trend (+ or -)	Z Value	Trend (+ or -)	Z Value
Robat	Yes (−)	−0.35	Yes (−)	−0.63
Cheshmeh-Khan	Yes (+)	**1.87**	Yes (+)	**1.76**
Tamor	Yes (+)	1.52	Yes (+)	3.75**
Bahlakeh-Dashli	Yes (+)	1.57	Yes (−)	−1.38
Arazkoseh	Yes (+)	1.48	Yes (−)	−1.24
Ramian	Yes (+)	3.18**	Yes (+)	1.44
Vashmgir	Yes (+)	2.45**	Yes (+)	4.38**
Tirtash	Yes (+)	3.67**	Yes (+)	2.86**
Agh Ghala	Yes (−)	−1.90*	Yes (−)	−2.22*
Ziarat	Yes (+)	4.17**	Yes (−)	−0.09
Fazel Abad	Yes (+)	2.32*	Yes (+)	3.15**
Ghafar Haji	Yes (+)	3.55**	Yes (+)	3.48**
Sefid Chah	Yes (+)	5.85**	Yes (+)	2.97**
Nozar Abad	Yes (+)	4.48**	Yes (+)	2.49**
Soleiman Tangeh	Yes (+)	0.44	Yes (+)	2.25*
Kareh Sang	Yes (+)	2.72**	Yes (+)	4.96**
Chamestan	Yes (+)	3.43**	Yes (+)	1.49
Pol-e-Zoghal	Yes (+)	2.44**	Yes (+)	**1.96**
Abas Abad	Yes (+)	2.25*	Yes (+)	4.63**
Gilvan	Yes (+)	0.58	Yes (+)	2.93**
Astaneh	Yes (+)	5.23**	Yes (+)	2.31*
Parodbar	Yes (+)	2.15*	Yes (−)	−1.96*
Manjil	Yes (+)	0.07	Yes (+)	3.39**
Rasht	Yes (+)	0.69	Yes (−)	−2.58**
Ghaleh Rudkhan	Yes (+)	5.16**	Yes (+)	2.10*
Kasma	Yes (+)	2.60**	Yes (+)	1.23
Masal	Yes (+)	4.06**	Yes (−)	−1.50
Astara	Yes (+)	3.80**	Yes (+)	3.06**
Mianeh	Yes (+)	1.31	Yes (−)	−0.32
Khalkhal	Yes (+)	5.28**	Yes (+)	1.61
Bandar Anzali	Yes (+)	3.46**	Yes (−)	−3.67**
Maraveh-Tappeh	Yes (+)	1.26	Yes (+)	1.40
Zarrineh	Yes (+)	3.83**	Yes (+)	3.79**
Sari	Yes (+)	2.19*	Yes (+)	2.35**
Ghorveh	Yes (+)	4.21**	Yes (+)	2.07*

Bold: Significance at 10 % level.

Bold & *: Significance at 5 % level.

Bold & **: Significance at 1 % level.

The precipitation has increased in the center of the study area and declined in the southwest and southeast of the SCS (Fig. 3c). However, in contrast to temperature and evapotranspiration, most of these trends have not been significant (Table 3). The precipitation has increased significantly in two (Siah Ab and Kelar Abad) and one (Shalman) (of 36) stations at 1 % and 5 % confidence levels, respectively, while for two stations (Gorgan and Siah Ab), it has decreased substantially at a 1 % confidence level. Further, it has declined significantly at the Razan and Talarom stations at 5 % and 10 % confidence levels, respectively.

**Table 3:** Results of the Mann-Kendall analysis of annual flow and precipitation. (NM: nonmonotonic).

Annual flow				Precipitation		
River name (Gauge name)	Trend (+ or -)	Z Value	ITA method	Gauge	Trend (+ or -)	Z Value
Atrak (Dashli borun)	Yes (−)	−0.15	NM (−)	Darband	Yes (−)	−0.84
Gorgan-Rud (Basir Abad)	Yes (−)	−3.58**	NM (−)	Til Abad	Yes (−)	−0.74
Kordkuy (Pole Jadeh)	Yes (−)	−0.72	NM (−)	Siah Ab	Yes (+)	2.61**
Baghoo (Baghoo)	Yes (+)	0.23	NM (+)	Talarom	Yes (−)	**−1.76**
Gaz (Vatana)	Yes (+)	0.72	NM (+)	Abloo	Yes (+)	0.04
Neka-Rud (Abloo)	Yes (−)	**−1.85**	NM (−)	Darabkola	Yes (+)	1.20
Darabkola (Darabkola)	Yes (−)	−0.26	NM (−)	Kord Khail	Yes (+)	0.73
Tajan (Kord Khail)	Yes (−)	−2.23*	NM (−)	Kiakola	Yes (−)	−0.44
Talar (Kiakola)	Yes (−)	−2.70**	NM (−)	Babol	Yes (+)	0.61
Babol-Rud (Babol)	Yes (−)	−1.18	NM (+)	Mian Dasht	Yes (+)	0.23
Haraz (Karehsang)	Yes (−)	−2.45**	NM (−)	Razan	Yes (−)	−2.28*
Lavij (Tangeh Lavij)	Yes (+)	1.16	NM (+)	Karehsang	Yes (+)	0.15
Chaloos (Pole Zoghal)	Yes (−)	−0.01	NM (+)	Pole Zoghal	Yes (+)	0.93
Sardab-Rud (Sardab-Rud)	Yes (+)	1.11	NM (+)	Kelar Dasht	Yes (−)	−2.66**
Palang-ab-Rud (Kelar Abad)	Yes (−)	0.00	NM (+)	Kelar Abad	Yes (+)	2.47**
Kazem-Rud (Mashaallah Abad)	Yes (+)	2.26*	NM (+)	Mashaallah Abad	Yes (+)	0.82
Cheshmeh kileh (Haratbar)	Yes (+)	0.49	NM (−)	Haratbar	Yes (+)	0.99
Chalek-Rud (Kangsar)	Yes (+)	0.15	NM (+)	Kangsar	Yes (−)	−0.70
Safa-Rud (Ramsar)	Yes (−)	−0.80	NM (−)	Ramsar	Yes (−)	−0.07
Pol-Rud (Derazlat)	Yes (−)	−0.93	NM (+)	Shalman	Yes (+)	2.07*
Shalamn-Rud (Shalman)	Yes (+)	0.13	NM (+)	Valt	Yes (+)	1.02
Azad-Rud (Dinar Sara)	Yes (−)	−0.23	NM (−)	Dinar Sara	Yes (+)	0.37
Khoshk-Rud (Bajigooabr)	Yes (−)	−1.51	NM (−)	Astaneh	Yes (+)	1.19
Alish-Rud (Oskoo Mahleh)	Yes (−)	−2.10*	NM (+)	Ghale Rood Khan	Yes (−)	−0.38
Tirom (Rezapet)	Yes (+)	2.12*	NM (−)	Kasma	Yes (−)	−1.20
Sefid-Rud (Astaneh)	Yes (−)	−3.25**	NM (−)	Shanderman	Yes (+)	0.08
Siah-Rud (Behdan)	Yes (−)	**−1.95**	NM (−)	Ghorveh	Yes (−)	−1.40
Chaf-Rud (Roodbar Sara)	Yes (−)	−1.40	NM (−)	Rasht	Yes (+)	0.16
Shafa-Rud (Poonel)	Yes (−)	−2.21*	NM (−)	Astara	Yes (+)	1.09
Nav-Rud (Kharjegil)	Yes (−)	−1.36	NM (−)	Bandar Anzali	Yes (−)	−0.38
Kargan-Rud (Mashin Khaneh)	Yes (−)	−1.10	NM (−)	Gorgan	Yes (−)	−3.38**
Shirabad (Shir Abad Bala)	Yes (−)	−1.29	NM (+)	Khalkhal	Yes (−)	−0.79
Choobar (Choobar)	Yes (+)	0.79	NM (+)	Maraveh Tappeh	Yes (+)	0.47
Landvil (Bash Mahaleh)	Yes (+)	0.68	NM (+)	Zarrineh	Yes (−)	−1.36
Ghasht-Rud (Pirsara)	Yes (−)	−0.05	NM (−)	Mianeh	Yes (−)	−0.89
Khalkaee (Tasko)	Yes (−)	−2.18*	NM (+)	Khalkhal	Yes (−)	−0.79
Morghak (Emamzade Shafy)	Yes (−)	−0.71	NM (−)			
Chelvand (Khan Hayati)	Yes (−)	−0.79	NM (+)			
Khaleh sara (Kaleh sara)	Yes (−)	−0.30	NM (−)			
Emamzade Ebrahim (Keshm)	Yes (+)	0.26	NM (−)			
Baharestan (Baharestan)	Yes (−)	**−1.89**	NM (−)			

Bold: Significance at 10 % level.

Bold & *: Significance at 5 % level.

Bold & **: Significance at 1 % level.

**Fig. 4:**
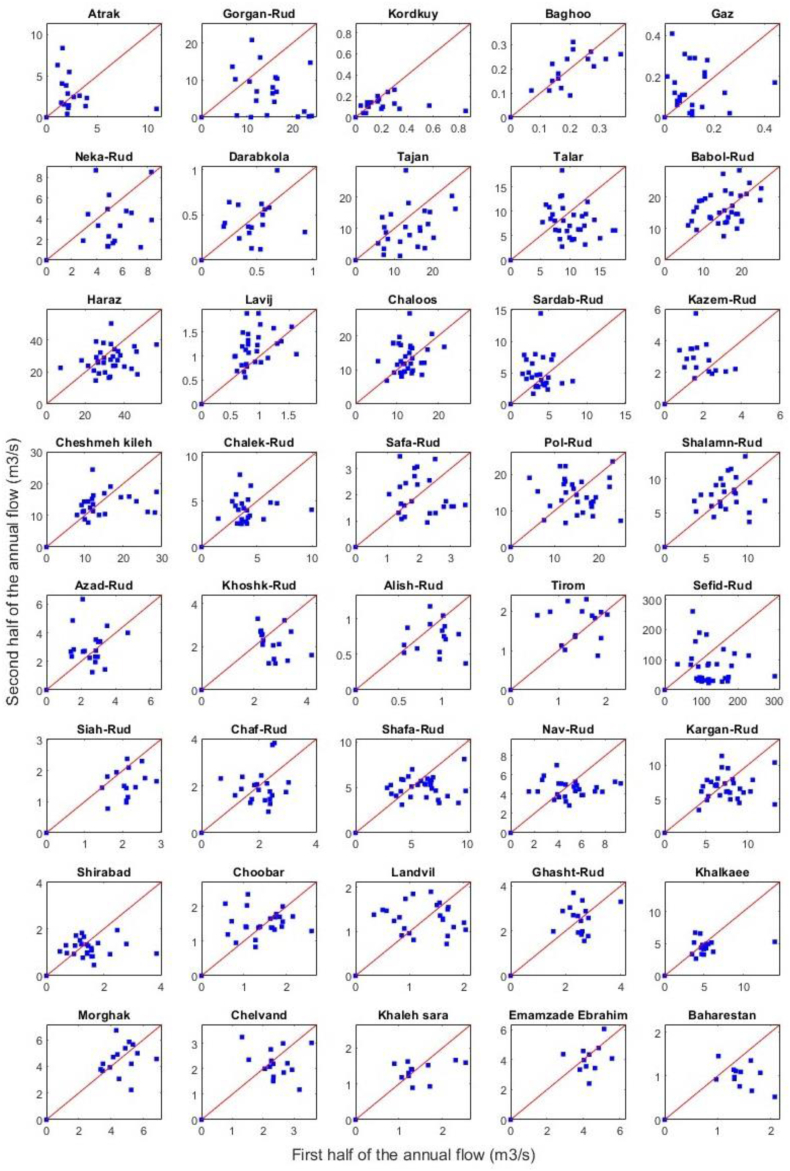
Results of the ITA method for the annual mean flow (The red line is 1:1 line). In each scatter diagram, the first half of the annual mean time series at a gauge has plotted against the second half.

The MK3 test Z-value for the annual mean flow has been negative in most of the gauges (28 of 40 gauges) (Fig. 3d), and annual mean flow in 5, 4, and 3 (of 40) gauges have declined substantially at 1 %, 5 %, and 10 % confidence level, respectively (Table 3). While the annual mean flow in the Tirom River at the Rezapet gauge and the Kazem-Rud at the Mashallah-Abad gauge increased significantly at a 5 % confidence level. Similarly, the ITA method results indicated that more than half gauges have a downward nonmonotonic trend (23 of 40 gauges) (Table 3). The trend in all gauges was nonmonotonic because the points in all the scatter diagrams have not entirely fallen above/below the 1:1 line and have intersected the line (Fig. 4).

### 3.2 The abrupt change in rivers’ flow and volume changes

In this study, the Pettitt test was applied to the available flow data (the whole period, which varied from 22 to 67 years (Fig. A1)) and three specific periods, including 1995–2017, 1988–2017, and 1978–2017 (Table 4).

**Table 4:** The results of the Pettitt test in various periods. The last column shows the results of the test in whole period of recorded data. The bold values indicate the significant abrupt change. All data is available up to the year 2017. (NA: not applicable).

River name	Gauge name	Length of data (year)	Periods			
			1995–2017	1988–2017	1978–2017	Whole period
Atrak	Dashli borun	31	2007	2007	NA	2007
Gorgan-Rud	Basir Abad	42	2006	**1998**	**1996**	**1995**
Kordkuy	Pole Jaddeh	42	2008	1998	1985	1998
Baghoo	Baghoo	34	2008	1989	NA	1986
Gaz	Vatana	47	**2006**	**2006**	2006	1986
Neka-Rud	Abloo	47	2000	2000	1982	1997
Darabkola	Darabkola	44	2005	2005	1986	1988
Tajan	Kord Khail	47	2007	**1998**	**1998**	**1988**
Talar	Kiakola	66	2007	1998	**1998**	**1998**
Babol-Rud	Babol	67	2007	**2007**	1987	1970
Haraz	Karehsang	67	2002	2002	2002	**1979**
Lavij	Tangeh Lavij	58	**2005**	**2005**	1987	**1987**
Chaloos	Pole Zoghal	67	2007	**1998**	2007	2007
Sardab-Rud	Sardab-Rud	51	2002	1996	1984	1984
Kazem-Rud	Mashaallah Abad	34	2001	2001	NA	**2001**
Cheshmeh kileh	Haratbar	47	2011	**1996**	1986	1986
Chalek-Rud	Kangsar	51	2007	2007	1983	2007
Safa-Rud	Ramsar	47	2001	1995	1988	1988
Pol-Rud	Derazlat	60	2011	1995	1980	1974
Shalamn-Rud	Shalman	49	2014	1994	1982	1982
Azad-Rud	Dinar Sara	39	2000	2000	2000	2000
Khoshk-Rud	Bajigooabr	31	2003	1998	NA	1998
Alish-Rud	Oskoo Mahleh	29	2005	**1998**	NA	**1998**
Tirom	Rezapet	29	1997	1995	NA	1995
Sefid-Rud	Astaneh	60	1998	**1998**	**1998**	**1996**
Siah-Rud	Behdan	29	**2003**	**2003**	NA	**2003**
Chaf-Rud	Roodbar Sara	42	2005	2005	2005	2005
Shafa-Rud	Poonel	60	2015	1994	1994	**1994**
Nav-Rud	Kharjegil	51	2005	1996	1996	1996
Kargan-Rud	Mashin Khaneh	60	2002	1994	1994	1994
Shirabad	Shir Abad Bala	49	2008	1994	1994	1994
Choobar	Choobar	47	1998	1998	1998	1998
Landvil	Bash Mahaleh	47	2008	1994	1994	1979
Ghasht-Rud	Pirsara	32	2005	2012	NA	2012
Khalkaee	Tasko	34	2005	1994	NA	1994
Morghak	Emamzade Shafy	32	1999	1994	NA	1994
Chelvand	Khan Hayati	31	2008	1999	NA	1999
Khaleh sara	Kaleh sara	31	2002	2002	NA	1994
Emamzade Ebrahim	Keshm	22	2010	NA	NA	2010
Baharestan	Baharestan	24	2005	NA	NA	**2005**

Considering the whole period, the detected abrupt change year varied from 1970 (Babol-Rud at the Babol gauge) to 2012 (Ghasht-Rud at the Pirsara gauge). By about 11 and 8 change points, the most detected years were between 1993–1997 and 1998–2002, respectively (Fig. A2d). Of 40 rivers, a significant abrupt change year occurred in 11 rivers varied from 1979 in the Haraz River (at the Karehsang gauge) to 2005 in the Baharestan River (at the Baharestan gauge) (Table 4). The most significant abrupt changes in the flow time series were detected in 6 and 4 rivers located in the center and west of the study area, respectively (Fig. 2e). In the center of the region, the most change points were detected from 1998 to 2002 while in the west, the most changes occurred between 1993 and 1997 (Fig. A3d).

From 1995 to 2017, the most change points were detected from 2003 to 2007, by about 18 abrupt change points of 40 rivers (Fig. A2a). In this period, the change point in three rivers was significant, including Gaz, Lavij, and Siah-Rud River at the Vatana, Tangeh Lavij, and Behdan gauges, respectively (Table 4). In the west and east of the study area, the most changes occurred between 2003 and 2007, while in the center, the most changes were detected from 1995 to 2002 (Fig. A3a).

In the other periods (1988–2017 and 1978–2017), the most detected years were between 1998 and 2002 (Figs. A2b and c). In these periods, a significant abrupt change was found in 10 (of 38) and 4 (of 27) river flow time series, most of which were detected from 1998 to 2002. The spatial distribution of the change points indicated that most of the changes in the west of the study area occurred between 1993 and 1997, while in the center, the most change points were observed between 1998 and 2002 (Figs. A3b and c).

**Fig. 5:**
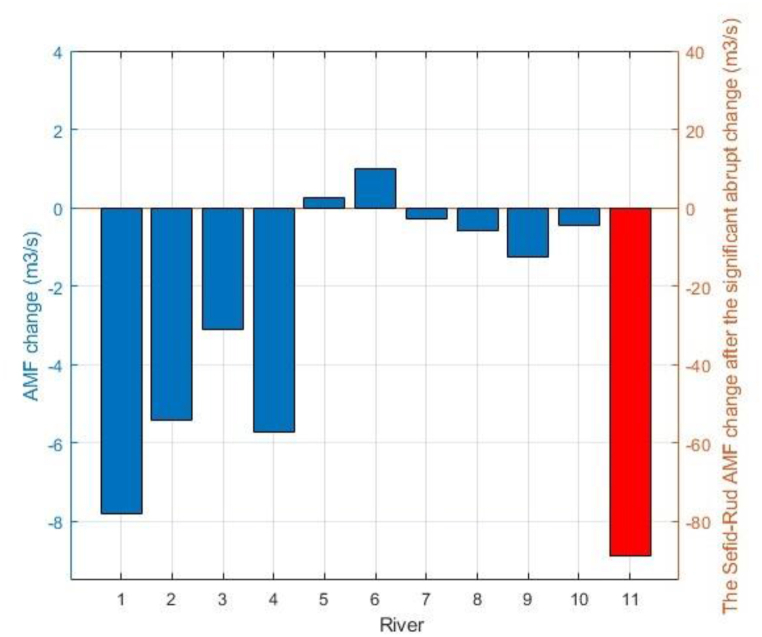
The mean annual flow (MAF) changes after the significant abrupt change in 1: 1: Gorgan-Rud at Basir Abad, 2: Tajan at Kord Khalil, 3: Talar at Kiakola, 4: Haraz at Karehsang, 5: Lavij at Tangeh Lavij, 6: Kazem-Rud at Mashallah Abad, 7: Alish Rud at Oskoo Mahalleh, 8: Siah-Rud at Behdan, 9: Shafa-Rud at Poonel, 10: Baharestan at Baharestan, and 11: Sefid-Rud at Astaneh. To clarify, the alteration in the Sefid-Rud River has been shown in the right y-axis and red color.

Considering the whole period, the AMF has declined in 9 (of 11) rivers after the significant abrupt change point that caused inflow to the Caspian Sea to decrease by about 3580 MCM annually. While the AMF has increased in two rivers located in the center of the study area, leading to an increase in inflow to the Sea by about 40 MCM annually. The most decline (increase) has occurred at the Astaneh gauge in the Sefid-Rud River (at the Mashaallah Abad in the Kazem-Rud River) where AMF has declined (increased) by about 88.9 (0.98) m^3^/s on average after the significant abrupt change in 1996 (2001) (Fig. 5).

### 3.3 Assessing the impacts of anthropogenic and climate change on rivers’ flow alteration

The results of elasticity-based methods on rivers with significant change points during the whole period indicate that anthropogenic activities played a dominant role in river flow alteration almost in all rivers (10 of 11 rivers), while in one river (Alish-Rud River), climate change played a key role. The relative contribution of anthropogenic activities to river flow alteration ranges from about 45.1 % (in the Alish-Rud River) to about 100 % in four rivers, including the Tajan, Talar, Lavij, and Kazem-Rud Rivers (on average of about 91.7 %). While the average relative contribution of climate change to river flow alteration was about 8.3 % (Table 5). Anthropogenic activities also played a pivotal role in river flow alteration in the other three periods, including 1995–2017, 1988–2017, and 1978–2017 by about 72.3 %, 79.5 %, and 94.3 % on average, respectively.

**Table 5:** The relative contribution of human activities and climate change on runoff change in the rivers with change point.

	River name	Gauge name	Sub-basin	Change point	Discharge change (m3/s)	Climate change (%)	Anthropogenic avtivities (%)
1995–2017	Gaz	Vatana		2006	−0.13	0.0	100
	Lavij	Tangeh Lavij	Lahijan-Noor	2005	−0.41	74.6	25.4
	Siah-Rud	Behdan	Talesh	2003	−0.68	8.5	91.5
1988–2017	Gorgan-Rud	Basir Abad	Gorgan-Rud	1998	−8.74	22.7	77.3
	Gaz	Vatana		2006	−0.13	0.0	100.0
	Tajan	Kord Khail	Haraz-Neka	1998	−7.92	2.9	97.1
	Babol-Rud	Babol		2007	−5.88	15.0	85.0
	Lavij	Tangeh Lavij	Lahijan-Noor	2005	−0.42	61.2	38.8
	Chaloos	Pole Zoghal	Lahijan-Noor	1998	−5.02	5.4	94.7
	Cheshmeh kileh	Haratbar	Lahijan-Noor	1996	−10.62	12.2	87.8
	Alish-Rud	Oskoo Mahleh	Lahijan-Noor	1998	−0.28	54.9	45.1
	Sefid-Rud	Astaneh	Sefid-Rud	1998	−105.02	27.1	72.9
	Siah-Rud	Behdan	Talesh	2003	−0.60	3.4	96.6
1978–2017	Gorgan-Rud	Basir Abad	Gorgan-Rud	1996	−8.62	12.6	87.4
	Tajan	Kord Khail	Haraz-Neka	1998	−5.64	0.0	100
	Talar	Kiakola	Haraz-Neka	1998	−3.03	0.0	100
	Sefid-Rud	Astaneh	Sefid-Rud	1998	−90.46	10.3	89.7
Whole period of recorded data	Gorgan-Rud	Basir Abad	Gorgan-Rud	1995	−7.79	9.5	90.5
	Tajan	Kord Khail	Haraz-Neka	1988	−5.42	0.0	100
	Talar	Kiakola	Haraz-Neka	1998	−3.10	0.0	100
	Haraz	Karehsang	Haraz-Neka	1979	−5.73	4.9	95.1
	Lavij	Tangeh Lavij	Lahijan-Noor	1987	0.26	0.0	100
	Kazem-Rud	Mashaallah Abad	Lahijan-Noor	2001	0.98	0.0	100
	Alish-Rud	Oskoo Mahleh	Lahijan-Noor	1998	−0.28	54.9	45.1
	Siah-Rud	Behdan	Talesh	2003	−0.60	3.4	96.6
	Shafa-Rud	Poonel	Talesh	1994	−1.27	0.4	99.6
	Baharestan	Baharestan	Talesh	2005	−0.45	9.7	90.3
	Sefid-Rud	Astaneh	Sefid-Rud	1996	−88.86	8.9	91.1

From a river flow decline point of view, anthropogenic activities played a dominant role in 8 rivers by about 95.4 %, on average, while in the Alish-Rud River, climate change played a pivotal role, accounting for 54.9 %. Anthropogenic activities by about 89.8 % led to inflow to the Caspian Sea decline by about 3216 MCM annually. In contrast, the mean contribution of climate change in river flow decline was approximately 10.2 %, which decreased inflow to the Sea by about 364 MCM yearly.

Anthropogenic activities have dramatically affected the AMF in the Lavij, and Kazem-Rud rivers by about 100 %, which led to inflow to the Caspian Sea has increased by about 39 MCM annually. In other periods, including 1995–2017, 1988–2017, and 1978–2017 anthropogenic activities have played a dominant role in river flow decline, which led to inflow to the Caspian Sea decreased by about 27.8 MCM, 3627 MCM, and 3203 MCM, after the change point, respectively.

## 4 Discussion

Precipitation and PET, representing available water and energy, respectively, are two key climate variables influencing the hydrological processes [56]. Although the precipitation has not changed significantly in the SCS (Table 3), the PET has soared in most of the study area, especially in the center of the region (Fig. 2b). It means that energy in the SCS has Increased, resulting from the increasing temperature (Table 2) that can lead to increasing actual evaporation. Other studies have reported increasing the evaporation rate in the study area [25]. On the other hand, increasing anthropogenic activities, including land use change and water withdrawal, have altered the rivers’ flow regime in the SCS and caused inflow to the Sea decrease by about 3540 MCM every year (Table 5). It will have wide-ranging implications for the livelihoods of the surrounding communities [26] and can accelerate the decreasing trend of the Caspian Sea level [24]. Decreasing the Caspian Sea level, resulting from declining inflow to the Sea, can accelerate eutrophication, leading to environmental degradation of the Caspian Sea that has inverse effects on the fishery industry, ecosystem service, and economic situation of people living on the Caspian Sea coast [23,57].

Dam construction in the SCS has been planned to meet water demand since 1962. The number of dams in the region dramatically increased from 1993 to 2007 (Fig. A4) and reached 117 in 2017. Along with the increase in the number of dams, the number of change points in the rivers has also increased (Fig. A2d and Fig. A4). By 2007, 95 dams had been commissioned, which regulate about 4.5 km^3^ annually (about 90 % of total regulated water across the sub-basins). These dams influence the inflow to the Caspian Sea considerably, particularly in the first decade of the 21st century. Although dam construction continued after 2007, the volume of newer dams was smaller than previous dams and did not significantly affect the river flow. Therefore, no significant abrupt change point was detected in the river flow time series after 2007 (Fig. A2).

In this study, the period of observed flow in the gauges differed (Fig. A1). Undoubtedly, the equal length of data can help investigate the flow alteration more accurately and decrease the degree of uncertainty. The change in AMF in three periods was analyzed by us to make a uniform and comparable assessment. The first period was 22 years (between 1995 and 2017), including all the gauges. This period could not adequately reflect all the changes in the SCS as the most anthropogenic activities had occurred between 1993 and 2002, e.g., the commissioning of 46 dams in this period (Fig. A4). Therefore, two more extended periods, including 30 and 40 years (i.e., 1988–2017 and 1978–2017), were considered to address flow regime alteration across the SCS. These periods covered more alterations in river flow time series, although the number of rivers was less than in the first period (38 and 27 rivers, respectively). The length of the flow's time series data can influence on detected change point by the Pettitt test, for example, shifting the time of the significant change point or the significance of the change point (Table 4). For example, when we applied the Pettitt test on the Chaloos River time series at the Pole Zoghal gauge from 1988 to 2017, a significant abrupt change was found in 1998. While in the next period (between 1978 and 2017), it was detected in 2007. Finally, when the test was applied to the time series at the gauge from 1950 to 2017 (representing the total length of the time series), a significant abrupt change was not detected. The detection of change points can be influenced by factors such as data distribution, outliers, and seasonality, occasionally resulting in the detection of multiple change points. Therefore, the careful selection of analysis periods is essential, considering the analysis goals, as longer time series can offer more information for change point detection.

The estimation of PET was one of the sources of uncertainty in the current study. Due to the lack of meteorological data in the study area, the modified Hargreaves model [34], which uses temperature data to calculate reference evapotranspiration, was applied. Therefore, the role of other meteorological variables was ignored in the PET estimation. Generally, PET is a complex nonlinear process, and a change in any one parameter can influence the other parameter(s) [58]. Although the effect of such changes on evapotranspiration is challenging to understand [59], applying some models such as FAO-PM 56 [60] which use more meteorological variables, including wind speed, humidity, and sunshine hours to estimate reference evapotranspiration would be very helpful to estimate it accurately and decrease uncertainty.

The elasticity-based methods were used to assess and quantify the impact of anthropogenic activities and climate change on river flow alteration. This approach with a realistic physical basis is the fastest method for this purpose and is easy to calculate [19]. In this approach, precipitation and temperature (for estimating PET) data were used to assess river flow alteration resulting from anthropogenic activities and climate change, with other variables' roles being disregarded. Furthermore, any alterations in precipitation and PET are imagined to be attributed to the impact of climate change. However human activities can exert direct influences such as urbanization, deforestation, and agricultural activities, as well as indirect impacts like greenhouse gas emissions and changes in land use, on precipitation and PET. Using physical-based hydrological modeling methods can reduce uncertainty in the results. However, these methods need a longer period of data, some of which are difficult to access or unavailable. In addition, these methods are costly, time-consuming, and complex approaches compared with elasticity-based methods.

The Iranian coastal regions, particularly Mazandaran, Golestan, and Gilan provinces, have witnessed a substantial increase in tourism-related GDP from 2005 to 2014. The growth rates in these provinces from 2011 onward have been 0.40 %, 1.01 %, and 1.33 %, respectively [61], with a concurrent urban population increase of 1.97 % between 2011 and 2016 [62]. Despite the potential for growth in rural areas, urbanization rates in Mazandaran and Gilan are lower compared to other provinces [63]. However, the surge in tourism has brought environmental challenges, including the loss of arable land and forests, increased household waste, and wastewater generation. A study in the Mazandaran region from 1990 to 2020 revealed significant changes in land use [64], contributing to pollution discharge into rivers during agricultural activities [65]. Despite these findings, coastal areas consistently maintain high rankings for cleanliness and are predominantly oligotrophic/mesotrophic [23].

Despite the negative environmental impacts, the fisheries sector in Iran faces challenges such as changes in hydrological regimes, sea level fluctuations, and pollution, resulting in a 15 % decrease in fish catch [62,66]. Although fisheries contribute only 0.4 % to the GDP, Iran remains a global leader in caviar and sturgeon meat exports [67]. The fisheries policy in Iran has shifted toward aquaculture since 2015, with the sector accounting for 35 % of total fisheries production [66,68]. Specific aquaculture initiatives, such as pen aquaculture in Gilan Province [69] and investments in rainbow trout farming in Mazandaran Province by Iran and the FAO, aim to address environmental concerns and boost the sustainability of fish production [70].

## 5 Conclusions

Quantitative analysis of meteorological variables using the MK3 and ITA approaches indicates an alarming trend of increasing temperature and PET and decreasing rivers’ flow in the SCS. This is evident in a decreasing nonmonotonic trend in most of the river flow in the region. The results of the Pettitt test during the whole period indicated that river flow altered significantly at 11 gauges leading to inflow to the Caspian Sea declining by about 3540 MCM on aggregate. Assessing and quantifying the impact of anthropogenic activities and climate change on river flow alteration in the SCS show that anthropogenic activities played a dominant role in river flow alteration in the SCS and have caused the volume of surface water flowing to the Sea decrease by about 3246 MCM every year, on aggregate. Furthermore, about 294 MCM of runoff decline was due to climate change in aggregate.

Given the attraction of the SCS for extended socio-economic activities along with the increasing trend of temperature, as the manifestation of climate change, supplying water needed for various sectors and its quality will be affected. Further, decreasing inflow to the Caspian Sea can accelerate the declining trend of the Sea level, which boosts eutrophication conditions in the Sea, and negatively affects the ecosystem and economics of the Caspian Sea. Therefore, an appropriate sustainable water resource management approach must be adopted in the SCS to ensure socio-economic activities can be continued.

## Data availability statement

The data will be available upon reasonable request.

## CRediT authorship contribution statement

**Alireza Sharifi:** Writing – original draft, Validation, Software, Methodology, Data curation, Conceptualization. **Aziza Baubekova:** Writing – review & editing. **Epari Ritesh Patro:** Writing – review & editing. **Bjorn Klove:** Writing – review & editing, Supervision. **Ali Torabi-Haghighi:** Writing – review & editing, Supervision, Methodology, Data curation, Conceptualization.

## Declaration of competing interest

The authors declare that they have no known competing financial interests or personal relationships that could have appeared to influence the work reported in this paper.
