# Supplementary material for: The combined effects of anthropogenic and climate change on river flow alterations in the Southern Caspian Sea Iran
Source: Heliyon. 2024 May 29;10(11):e31960. doi: 10.1016/j.heliyon.2024.e31960 (PMC11176795; doi:10.1016/j.heliyon.2024.e31960)
Supplement: Multimedia component 1 [file mmc1.docx]

**Supplementary materials of “The combined effects of anthropogenic and climate change on river flow alterations in the Southern Caspian Sea Iran”**


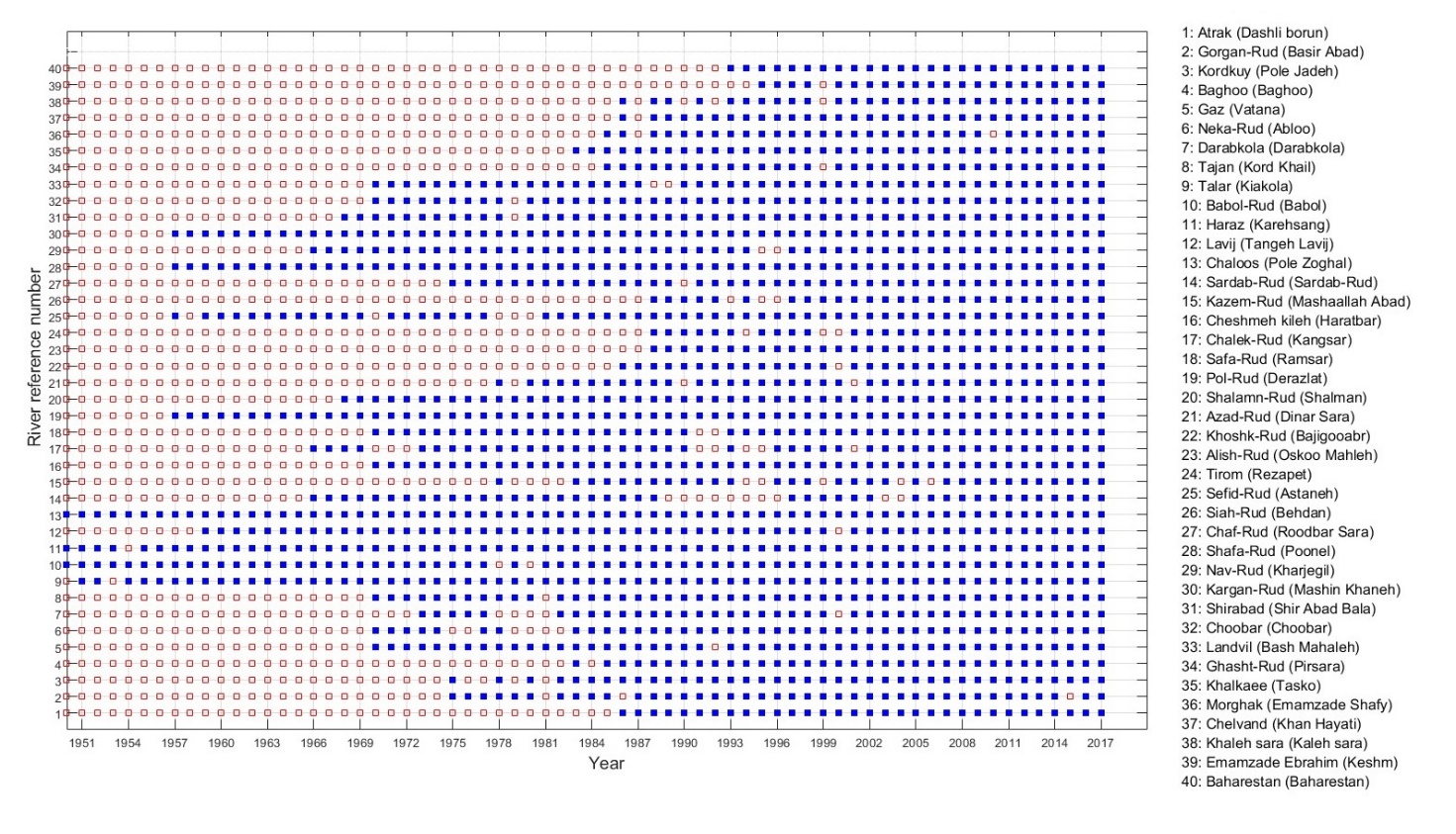


**Figure SI1**: The length of recorded flow data in the gauges until 2017. The blue and red squares show recorded and unrecorded flow data in a gauge, respectively.


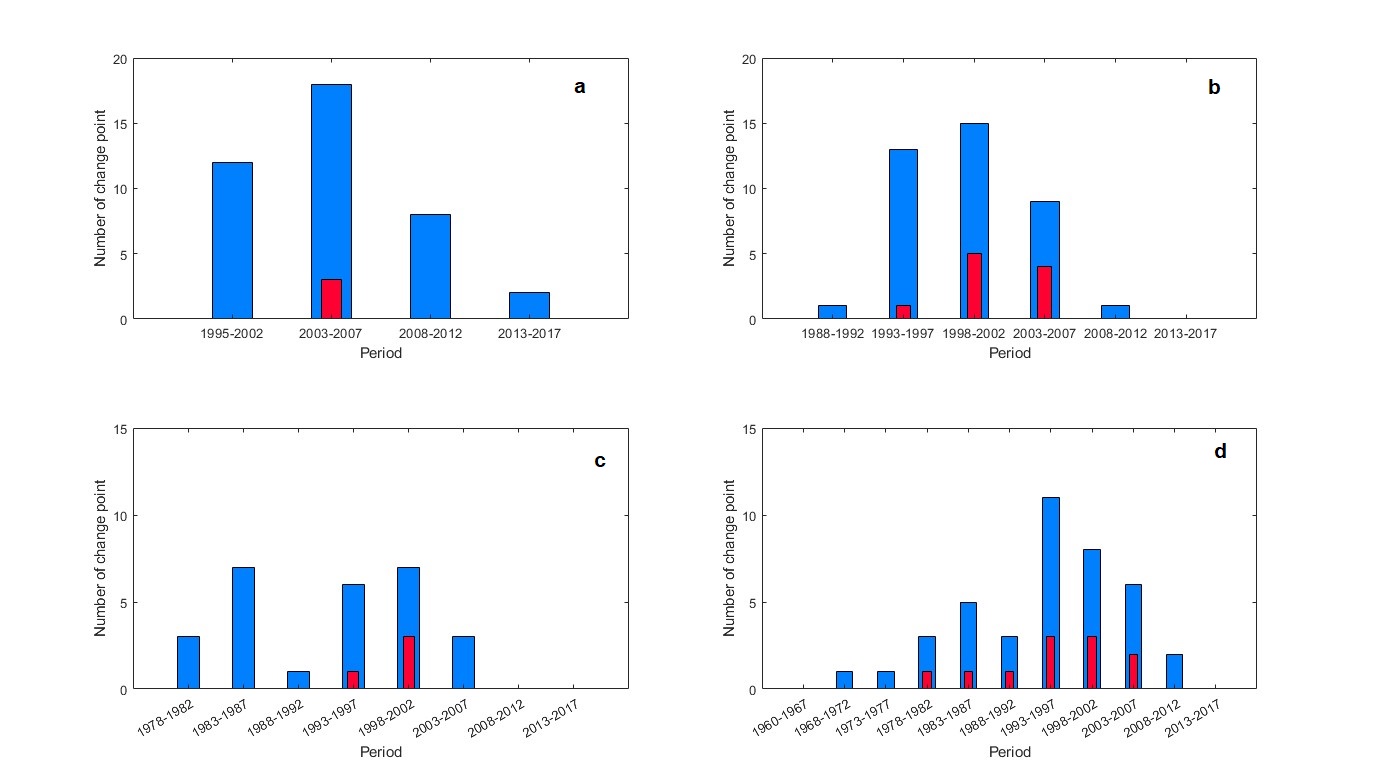


**Figure SI2**: The number of change points obtained from the Pettitt test in the rivers in various periods, including a:1995-2017, b: 1988-2017, c: 1978-2017, and d: the whole period of recorded data. The red bar shows the number of significant change points in the rivers.


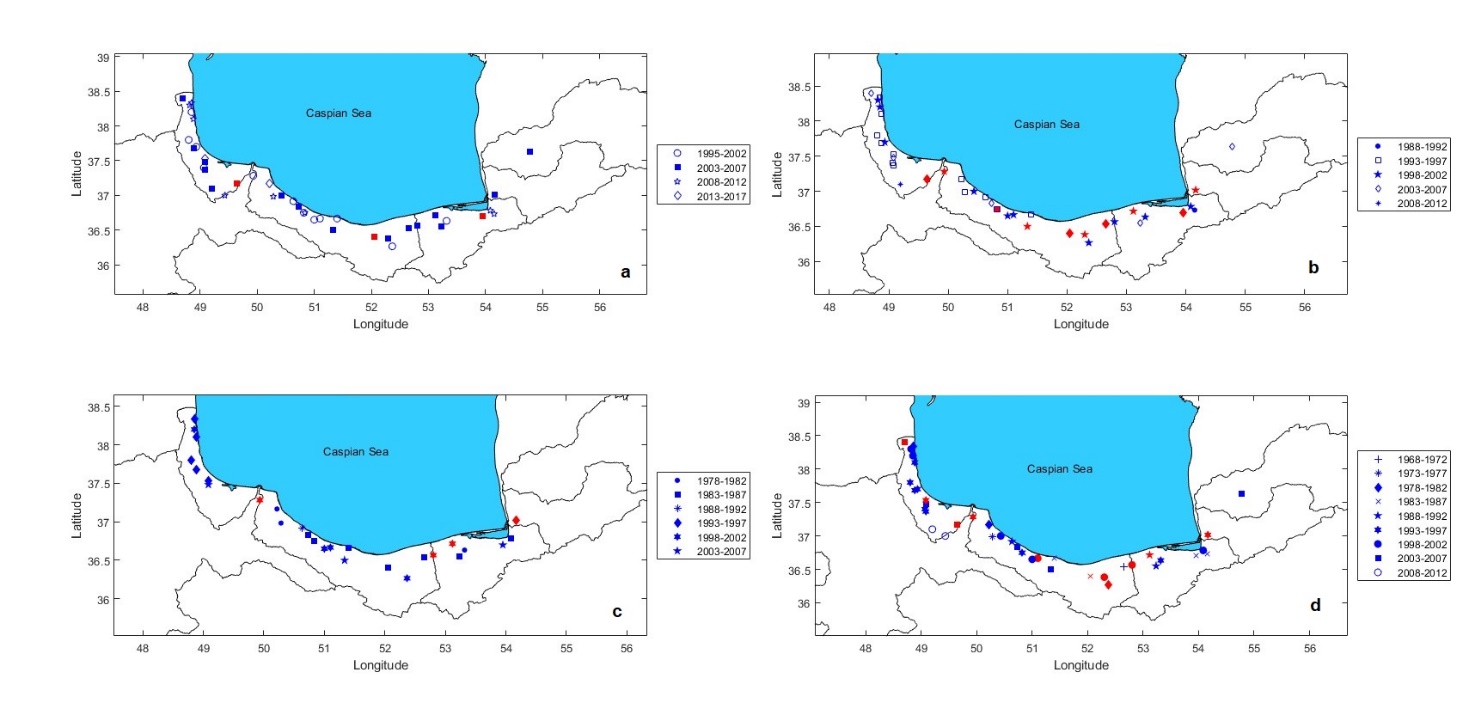


**Figure SI3**: The spatial distribution of change points obtained from the Pettitt test in various periods, including a:1995-2017, b: 1988-2017, c: 1978-2017, and d: the whole period of recorded data. The red marker shows the significant change point.


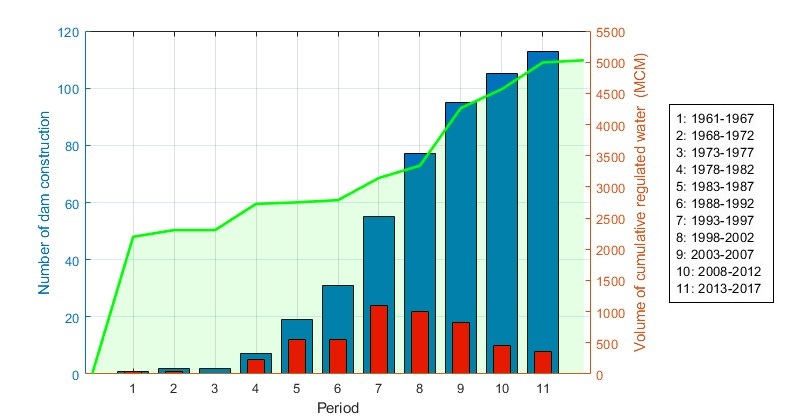


**Figure SI4**: The number of dams and the cumulative regulated water volume in the southern Caspian Sea from 1961 to 2017. The red and blue bar charts show the number of dams and the cumulative number of dams, respectively (left y-axis). Also, the green line shows the cumulative regulated water by the dams (right y-axis).
